# Prognostic Value of Pretreatment Lymphocyte-to-Monocyte Ratio and Development of a Nomogram in Breast Cancer Patients

**DOI:** 10.3389/fonc.2021.650980

**Published:** 2021-12-17

**Authors:** Ying Yin, Yong Zhang, Li Li, Shaotong Zhang, Ning Liu, Shuanghu Yuan

**Affiliations:** ^1^ Clinical Medical College, Southwest Medical University, Luzhou, China; ^2^ Department of Radiation Oncology, Shandong Cancer Hospital and Institute, Shandong First Medical University and Shandong Academy of Medical Sciences, Shandong Cancer Hospital Affiliated to Shandong First Medical University, Jinan, China; ^3^ Department of Radiation Oncology, Rongcheng People's Hospital, Rongcheng, China; ^4^ Department of Ultrasound, Jinan Central Hospital Affiliated to Shandong First Medical University, Jinan, China; ^5^ Department of Radiation Oncology, Shandong Cancer Hospital Affiliated to Shandong University, Jinan, China; ^6^ Department of Radiation Oncology, The Affiliated Cancer Hospital of Zhengzhou University, Henan Cancer Hospital, Zhengzhou, China

**Keywords:** lymphocyte-to-monocyte ratio (LMR), nomogram, disease-free survival (DFS), hematologic parameters, breast cancer

## Abstract

**Purpose:**

The objective of this study was to explore the prognostic significance of pretreatment hematologic parameters in predicting disease-free survival (DFS) of breast cancer patients.

**Materials and Methods:**

The medical records of 440 breast cancer patients in Shandong Cancer Hospital and Institute from 2003 to 2013 were analyzed retrospectively. Through the results of blood routine before treatment, the absolute lymphocyte count (ALC), absolute neutrophil count (ANC), absolute monocyte count (AMC), and absolute platelet count (APC) in peripheral blood were collected. The lymphocyte-to-monocyte ratio (LMR), neutrophil-to-lymphocyte ratio (NLR), platelet-to-lymphocyte ratio (PLR), and neutrophil-to-monocyte ratio (NMR) were calculated. Cox proportional hazard model was used for univariate and multivariate analysis. The DFS was compared using Kaplan–Meier method. The prognostic nomogram of patients with breast cancer was developed.

**Results:**

The median DFS for all patients was 64.10 months. Univariate analysis showed that the DFS was associated with surgical approach, TNM stage, molecular subtype, neoadjuvant chemotherapy, radiotherapy, and LMR (*p* < 0.05). TNM stage, molecular subtype, and LMR were independent prognostic factors of breast cancer in multivariate analysis (*p* < 0.05). According to the Kaplan–Meier survival curve analysis, patients with higher LMR (≥4.85) were associated with longer median DFS (median DFS, 85.83 vs. 60.90, *p* < 0.001). The proposed nomogram that incorporated LMR, TNM stage, and molecular subtype got a concordance index (c-index) of 0.69 in predicting 5-year DFS.

**Conclusion:**

In breast cancer patients, higher LMR was associated with longer median DFS and the nomogram including LMR, TNM stage, and molecular subtype could accurately predict the prolonged 5-year DFS of breast cancer patients.

## Introduction

Breast cancer is the most common cancer in women; patients in China account for 12.2% of all newly diagnosed breast cancers and 9.6% of all deaths from breast cancer around the world (1). Although the number of deaths from breast cancer has declined due to the improved cancer treatment, 20% to 30% patients still experience recurrence and distant metastases (2, 3). Therefore, it is particularly important to screen patients with higher recurrence and metastasis early for individualized treatment of breast cancer.

Pathological indicators such as tumor size, axillary lymph node metastasis, and histological grade, as well as molecular biological indicators such as estrogen receptor (ER), progesterone receptor (PR), human epidermal growth factor receptor-2 (HER-2), and ki-67 are currently used to guide the prognosis of breast cancer patients (4). These indicators mainly obtained by biopsy or postoperative pathological reports are difficult to represent the overall condition of the tumor. Consequently, it is necessary to find a non-invasive, comprehensive, and preoperative index to assess the prognosis of breast cancer patients. There is growing evidence supporting the role of inflammation in cancer development, progression, metastasis, and treatment of drug resistance, and the changes of tumor-related inflammatory cells reflect the degree of tumor inflammatory response (5, 6). Based on the number of circulating inflammatory cells, some hematological parameters have been recommended as simple parameters for the assessment of systemic inflammation and have been associated with the prognosis of various cancers (7, 8). Commonly used hematological parameters include absolute lymphocyte count (ALC), absolute neutrophil count (ANC), absolute monocyte count (AMC), absolute platelet count (APC), lymphocyte-to-monocyte ratio (LMR), neutrophil-to-lymphocyte ratio (NLR), platelet-to-lymphocyte ratio (PLR), and neutrophil-to-monocyte ratio (NMR). At present, studies have also linked hematological parameters to the prognosis of breast cancers (9–13). However, no evidences showed the prognostic values of systematic hematological parameters and clinical information in breast cancer.

Therefore, we comprehensively compared the eight pretreatment hematological parameters (i.e., ALC, ANC, AMC, APC, LMR, NLR, PLR, and NMR) and clinical features, in order to find the most useful hematological parameters to accurately predict the disease-free survival (DFS) of breast cancer patients.

## Materials and Methods

### Patient Selection

Patients with newly diagnosed primary breast cancer were retrospectively collected from 2003 to 2013 in Shandong Cancer Hospital and Institute. The inclusion criteria were as follows: (1) patients with accurate pathological diagnosis, complete blood routine, and clinicopathological data; (2) patients with TNM stages 0 to III; and (3) patients who have undergone immunohistochemistry (IHC) and/or fluorescence *in situ* hybridization (FISH). The exclusion criteria were as follows: (1) patients treated with glucocorticoids, sex hormones, and other drugs affecting blood routine outcomes 8 weeks before treatment; (2) patients with liver and kidney diseases, blood system diseases, and other diseases that affect peripheral hematologic indicators; (3) patients with a history of blood transfusion 1 week prior to treatment; and (4) patients with inflammatory breast cancer. Finally, 440 patients were eligible for analysis and were reviewed retrospectively. All the enrolled patients underwent surgery and followed standard treatment guidelines as outlined during that time frame in our institution. Take the time of pathological diagnosis as the starting point of observation, every patient was followed up regularly by outpatient revisit and telephone call until progressive disease (PD) or until death, and the last follow-up date is June 23, 2019.

### Data Collection

Patient characteristics and hematologic data were obtained from electronic medical records from Shandong Cancer Hospital and Institute. Target variables include basic clinical features, treatment, PD, and pretreatment hematological parameters. The basic clinical features included age, menopausal status, family history, location, TNM stage, and molecular subtype; treatment included surgical approach, radiotherapy, and neoadjuvant chemotherapy; PD was defined as local recurrence or distant metastasis; pretreatment hematological parameters included ALC, ANC, AMC, APC, LMR, NLR, PLR, and NMR. Molecular subtype was carried out according to the St Gallen-2013 recommendations. A tumor was considered luminal A-like if it had positive ER and PR, HER-2 negative, and Ki-67 low. A tumor was classified as luminal B-like if (1) it had positive ER, HER-2 negative, and Ki-67 high or negative PR or (2) positive ER and positive HER-2. A tumor was considered HER-2 positive (non-luminal) if it was ER and PR negative and HER-2 positive. Finally, tumors with ER, PR, and HER-2 negative were classified as triple-negative breast cancer (TNBC). The staging was based on the 7th edition of the staging system of the American Joint Committee on Cancer (AJCC).

### Statistical Analysis

DFS was calculated as the time between pathological diagnosis and the progression of the disease or death from breast cancer. All statistical analyses were performed using SPSS version 23.0 software (IBM, USA). The cutoff points for the continuous variables were based on the median value of each factor. Cox proportional hazard model was used for univariate and multivariate analyses. The multivariate analysis method was the forward LR method. Kaplan–Meier method was used to compare DFS, and log-rank test was used to compare survival curves. A nomogram for possible prognostic factors was formulated to provide visualized risk prediction using R software with the survival and rms packages. The performance of the nomogram for predicting survival was evaluated with Harrell’s concordance index (c-index), which is a measure of discrimination. The c-index > 0.5 indicates that the model could discriminate the outcome. Calibration of the nomogram for 5-year DFS was performed by comparing the predicted outcomes with the observed outcomes. *p*-values reported were bidirectional, and the significant level was at <0.05.

## Results

### Patient Characteristics and Hematologic Parameters

In total, 440 patients with breast cancer were enrolled in this study. All patients were female with a median age of 48 (19–81) years at the time of diagnosis. Of the 440 patients, 49 (11.1%) underwent breast conserving surgery and 391 (88.9%) underwent radical mastectomy. Of the 440 patients, 231 (52.5%) received endocrinotherapy, 115 (26.1%) received neoadjuvant therapy, and 205 received radiotherapy (46.6%). The other baseline clinicopathological data are shown in **Table 1**. The baseline mean values for ALC, AMC, ANC, APC, NLR, LMR, PLR, and NMR were 1.83 ± 0.55 (×10^9^/L), 0.41 ± 0.36 (×10^9^/L), 3.82 ± 1.46 (×10^9^/L), 259.12 ± 198.02 (×10^9^/L), 2.28 ± 1.27, 5.18 ± 2.84, 152.71 ± 121.07, and 10.97 ± 8.03, respectively.

**Table 1:** Baseline characteristics of the enrolled patients.

Variables	No. (%)
**Age (years)**	
<48	205 (46.6)
≥48	235 (53.4)
**Menopausal state**	
Menopause	154 (35)
Premenopause	286 (65)
**Family history**	
No	421 (95.7)
Yes	19 (4.3)
**Location**	
Left	258 (58.6)
Right	182 (41.4)
**Surgical approach**	
Breast conserving surgery	49 (11.1)
Mastectomy	391 (88.9)
**TNM stage**	
0	7 (1.6)
I	69 (15.7)
II	203 (46.1)
III	161 (36.6)
Molecular subtype	
Luminal A	101 (23)
Luminal B	193 (44)
HER-2	72 (16.4)
TNBC	73 (16.6)
**Neoadjuvant chemotherapy**	
Yes	115(26.1)
No	325(73.9)
**Radiotherapy**	
Yes	205 (46.6)
No	235 (53.4)

HER-2, human epidermal growth factor receptor-2; TNBC, triple-negative breast cancer.

### Univariate and Multivariate Analyses of DFS

**Figure 1:**
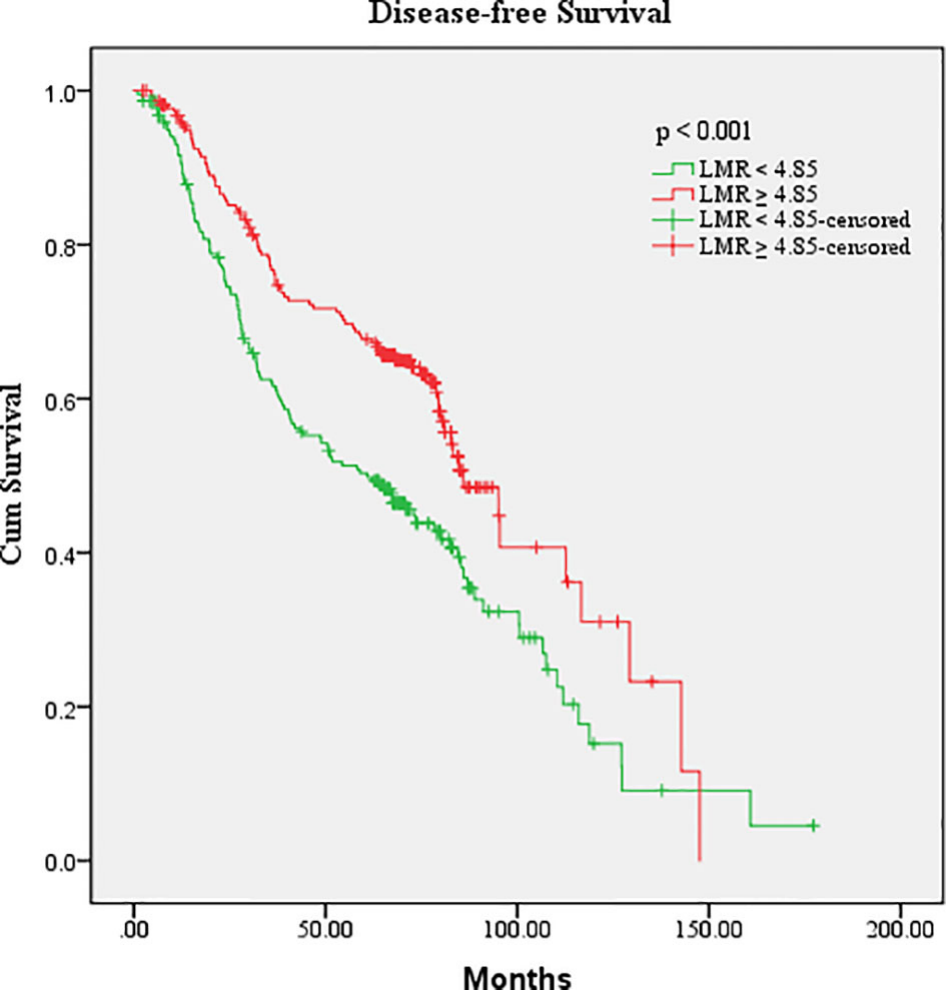
Kaplan–Meier estimates of the DFS of breast cancer patients according to the LMR level in overall patients.

After a median follow-up of 72.9 months, 224 (51%) had disease progression and 62 (14%) died among the 440 patients. The median DFS for all patients was 64.1 months. The cutoff points for the hematologic parameters were based on the median value of each factor, which were AMC [0.37×10^9^/L (range, 0.03–7.10×10^9^/L)], ANC [3.62×10^9^/L (range, 1.21–10.32×10^9^/L)], ALC [1.78×10^9^/L (range, 0.57–4.80×10^9^/L)], APC [239.00×10^9^/L (range, 29–2,302×10^9^/L)], NLR [2.00 (range, 0.48–12.79)], LMR [4.85 (range, 0.68–39.25)], PLR [132.33 (range, 17.24–1,487.84)], and NMR [9.68 (range, 0.44–120.67)]. Univariate analysis showed that the DFS of patients with breast cancer was associated with surgical approach (hazard ratio [HR]: 2.000, 95% confidence interval [CI]: 1.164–3.439), TNM stage (HR: 2.202, 95% CI: 1.786–2.715), molecular subtype (HR: 1.240, 95% CI: 1.093–1.406), neoadjuvant chemotherapy (HR: 0.511, 95% CI: 0.386–0.677), radiotherapy (HR: 0.700, 95%CI: 0.537–0.911), and LMR (HR: 0.607, 95% CI: 0.464–0.794) (**Table 2**). ALC, ANC, AMC, APC, NLR, PLR, and NMR did not show any association with DFS (*p* > 0.05). The above indexes related to DFS were included in multivariate analysis, and the results showed that TNM stage (*p* < 0.001), molecular subtype (*p* = 0.001), and LMR (*p* = 0.004) were independent prognostic factors of breast cancer (**Table 2**). Kaplan–Meier survival curve analysis showed that higher LMR (≥4.85) was associated with longer median DFS (median DFS, 85.83 vs. 60.90, *p* < 0.001) (**Figure 1**).

**Table 2:** Univariate and multivariate Cox regression analyses of hematologic parameters and clinicopathological characteristics for survival in patients with breast cancer.

Variables	Disease-free survival			
	Univariate analysis		Multivariate analysis	
	HR (95% CI)	*p*	HR (95% CI)	*p*
Age	1.081 (0.831–1.407)	0.561		
Menopausal state	0.810 (0.615–1.086)	0.135		
Family history	0.559 (0.248–1.259)	0.161		
Location	1.033 (0.791–1,350)	0.809		
Surgical approach	2.000 (1.164–3.439)	0.012		
TNM stage (reference 0)	2.202 (1.786–2.715)	<0.001		<0.001
I			1.205 (0.403–3.606)	0.739
II			1.538 (0.552–4.287)	0.410
III			4.776 (1.704–13.386)	0.003
Molecular subtype (reference Luminal A)	1.240 (1.093–1.406)	0.001		0.010
Luminal B			1.717 (1.171–2.517)	0.006
HER-2			1.987 (1.258–3.140)	0.003
TNBC			1.954 (1.244–3.070)	0.004
Neoadjuvant chemotherapy	0.511 (0.386–0.677)	<0.001		
Radiotherapy	0.700 (0.537–0.911)	0.008		
AMC (<0.37 vs. ≥0.37 × 10^9^/L)	1.220 (0.934–1.593)	0.145		
ANC (<3.62 vs. ≥3.62 × 10^9^/L)	1.152 (0.886–1.498)	0.292		
ALC (<1.78 vs. ≥1.78 × 10^9^/L)	0.886 (0.681–1.153)	0.368		
APC (<239 vs. ≥239 × 10^9^/L)	1.302 (0.998–1.699)	0.052		
NLR (<2.00 vs. ≥2.00)	1.280 (0.983–1.666)	0.067		
LMR (<4.85 vs. ≥4.85)	0.607 (0.464–0.794)	<0.001	0.671 (0.511–0.881)	0.004
PLR (<132.33 vs. ≥132.33)	1.070 (0.823–1.392)	0.614		
NMR (<9.68 vs. ≥9.68)	0.981 (0.751–1.281)	0.887		

AMC, absolute monocyte count; ANC, absolute neutrophil count; ALC, absolute lymphocyte count; APC, absolute platelet count; NLR, neutrophil-to-lymphocyte ratio; LMR, lymphocyte-to-monocyte ratio; PLR, platelet-to-lymphocyte ratio; NMR, neutrophil-to-monocyte ratio; HER-2, human epidermal growth factor receptor-2; TNBC, triple-negative breast cancer.

### Nomogram for the Prediction of DFS

**Figure 2:**
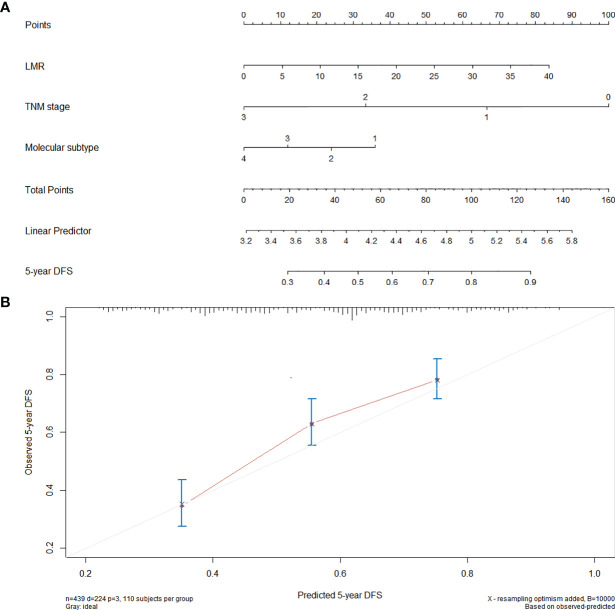
Nomogram for the prediction of DFS. **(A)** A nomogram for predicting the 5-year DFS for 440 patients with breast cancer (molecular subtype: 1 = luminal A, 2 = luminal B, 3 = HER-2, 4 = TNBC). To calculate the 5-year DFS for a specific patient, locate patient’s pretreatment LMR and draw a line straight upward to the Points axis to determine the score associated to that LMR. Repeat the process for TNM stage and molecular subtype, sum the scores, and locate this sum on the Total Points axis. Then, draw a line straight down to the corresponding “5-year DFS” axis to find the predicted 5-year DFS. **(B)** Calibration curves for 5-year DFS using nomograms with TNM stage, molecular subtype, and pretreatment LMR are shown. The *x*-axis is nomogram predicted probability of survival and *y*-axis is actual survival. The bootstrapping method was used for the internal validation of the nomogram. The gray line indicates perfect calibration.

A prognostic nomogram was established through Cox regression model analysis according to all significant independent indicators of DFS (i.e., TNM stage, molecular subtype, and LMR) (**Figure 2A**). Each factor in the nomogram was assigned a weighted number of points, and the sum of points for each patient was in accordance with a specific predicted 5-year DFS. For internal validation, the bootstrapped calibration plot of the nomogram predicting 5-year DFS performed well with the ideal model (**Figure 2B**). The c-index of the model was 0.69 (95% CI: 0.65–0.73).

## Discussion

This study comprehensively evaluated the predictive value of eight hematological parameters for DFS in breast cancer patients and found that only LMR was related to DFS, and the high LMR group had longer DFS. The nomogram including LMR, TNM stage, and molecular subtype could accurately predict the 5-year DFS of breast cancer patients.

Some previous studies have confirmed that high pretreatment LMR tended to be correlated with better survival for lung cancer, gastric cancer, and colorectal cancer (14–16), and our study also showed that higher LMR was associated with longer median DFS in breast cancer patients. However, the mechanism explaining the relationship between a high LMR and superior outcome in these cancers remains unclear. As an important part of inflammatory response, mononuclear macrophages release cytokines, create a chronic oxidative stress environment, and generate free radicals related to angiogenesis, tumor cell invasion, and metastasis, thus promoting tumor progression (17). In addition, monocytes have been proven to inhibit T lymphocyte proliferation and activation, leading to immunosuppression, thereby suppressing immune attacks against tumor cells (18). The increase in circulating monocytes may be used as a surrogate marker for high tumor burden (10, 19) and is associated with poor prognosis (20). On the other hand, lymphocytes are one of the key factors in immune surveillance and immune editing, which play the role of anti-tumor immunity by inducing tumor cell apoptosis, thus inhibiting the proliferation and migration of tumor cells (21, 22). A number of studies have also shown that low lymphocyte counts may lead to insufficient immune response, leading to low survival rates for many cancers (23, 24). Based on the reasons mentioned above, it is reasonable that the LMR combining the information of lymphocytes and monocytes is a potential indicator of prognosis for estimating the outcome of individuals, and a higher LMR may indicate a stronger antitumor capacity and may be relevant to a longer DFS.

In this study, in addition to LMR, TNM stage and molecular subtype were also independent predictors of DFS in breast cancer patients. It is widely known that postoperative TNM stage is the important factor affecting the prognosis of breast cancer patients, and the median 5-year survival was as follows: stage I, 97%, stage II, 78%, stage III, 52%, and stage IV, 13% (25). Breast cancer is a heterogeneous disease consisting of several molecular subtypes that have different biological behavior, different response to local and systemic treatment, and different prognosis (26). Luminal subtypes tend to have a better prognosis than non-luminal subtypes since luminal subtypes are hormone-receptor-positive and therefore more sensitive to hormone therapy (27, 28). The prognosis of HER-2 positive and TNBC is relatively poor, and they are more prone to early and frequent recurrence and metastasis. The HER-2-positive subtype has a superior prognosis to the TNBC because it could be treated with trastuzumab (29). Therefore, TNM stage and molecular subtype are important factors influencing DFS in breast cancer patients.

In order to make the results more readable and facilitate patient assessment, we combined clinicopathological factors and LMR to establish a nomogram to predict 5-year DFS of breast cancer patients through Cox regression model analysis. The 5th year after surgery was a peak period of recurrence and metastasis of breast cancer patients (30). So, predicting the 5-year survival rate of patients has important clinical significance. Nomogram is an important statistical model (31), which was used to predict survival for many cancers and has been recognized as superior to traditional TNM staging systems (32–34), and the estimates based on multivariate models are more reliable than single risk factor (35). In this study, the internal verification of the nomogram including LMR, TNM stage, and molecular subtype showed good discrimination (c-index, 0.69), and it could also be well calibrated to predict 5-year DFS. This practical model that combined clinicopathological factors and hematological parameters could help clinicians better predict DFS of breast cancer patients.

However, this study had several limitations. First, it is a single-center retrospective study with a relatively small-sized sample. Second, it lacks an external validation cohort, which could further confirm its robustness beyond the present data. Third, the hematologic parameters are non-specific biomarkers that may be affected by various pathophysiologic conditions and thus will vary from time to time. In this study, we mainly focused on the correlation between baseline hematologic parameters and DFS to aid in the optimal individualized management of patients with breast cancer. Further prospective multi-center studies are needed to determine the advantages and disadvantages of these results.

## Conclusion

To conclude, in breast cancer patients, higher LMR was associated with longer median DFS, and the nomogram containing LMR, TNM stage, and molecular subtype accurately predicted 5-year DFS.

## Data Availability Statement

The raw data supporting the conclusions of this article will be made available by the authors, without undue reservation.

## Author Contributions

Study design: SY; Data acquisition and analysis: YY, YZ; Interpretation of the data: LL, SZ, NL; Drafting of the manuscript: YY; Revision of the manuscript: SY. All authors contributed to the article and approved the submitted version.

## Funding

This study was partially funded by the Natural Science Foundation of China (NSFC81872475 and NSFC82073345).

## Conflict of Interest

The authors declare that the research was conducted in the absence of any commercial or financial relationships that could be construed as a potential conflict of interest.

## Publisher’s Note

All claims expressed in this article are solely those of the authors and do not necessarily represent those of their affiliated organizations, or those of the publisher, the editors and the reviewers. Any product that may be evaluated in this article, or claim that may be made by its manufacturer, is not guaranteed or endorsed by the publisher.
